# Applications of multislice CT angiography in the surgical clipping and endovascular coiling of intracranial aneurysms^☆^

**DOI:** 10.1016/S1674-8301(10)60062-0

**Published:** 2010-11

**Authors:** Wenhua Chen, Yilin Yang, Wei Xing, Ya Peng, Jianguo Qiu, Zhongming He, Qi Wang

**Affiliations:** a Department of Radiology,; b Departments of Neurosurgery, the Third Affiliated Hospital of Soochow University, Changzhou, Jiangsu 213003, China

**Keywords:** intracranial aneurysm, computed tomography angiography, clipping, coiling

## Abstract

Prompt diagnosis and therapy of aneurysms are critical for patients with nontraumatic subarachnoid hemorrhage (SAH). The aim of our study was to assess the clinical usefulness of multislice computed tomography angiography (CTA) in the surgical and endovascular treatment of intracranial aneurysms. A total of 195 cases with 206 intracranial aneurysms underwent CTA. Fifty (24%) aneurysms underwent surgical clipping while 156 (76%) aneurysms underwent endovascular coiling. In the five missed aneurysms at digital substraction angiography and the nine aneurysms with mass intracerebral hematomas, surgical treatment was successfully performed based on 16-slice CTA alone, and the other 36 aneurysms were clipped on the main basis of the CTA. The intraoperative findings correlated well with the CTA findings and all aneurysms were clipped successfully. Sixteen-slice CTA image information has been shown to determine the choice of aneurysm therapy and assist the surgical and endovascular treatment of intracranial aneurysms.

## INTRODUCTION

Nontraumatic subarachnoid hemorrhage (SAH) is a neurological emergency characterized by extravasation of blood into the spaces lining the central nervous system, which are normally filled with fluid. The main cause of nontraumatic SAH (80% of cases) is rupture of an intracranial aneurysm, an event accompanied by high morbidity and mortality rates[1]-[3]. Severe SAH rebleeding and death rates may be minimized by adequate neurosurgical or endovascular treatment[4]-[6]. Therefore, prompt diagnosis and therapy of the aneurysms are critical for patients with nontraumatic SAH. Computed tomography angiography (CTA) is a noninvasive volumetric imaging technique. Imaging acquisition of CTA requires only 1 min or less, and CTA is well tolerated by the majority of high-risk patients with acute SAH. CTA can be performed after plain CT and, if positive, it improves the prognosis of SAH because it provides an early diagnosis[7]-[10]. Moreover, a positive CTA result guides the treated procedure, which can be either neurosurgical treatment of the aneurysm or endovascular therapy[7],[11]-[13].

Many reports put particular emphasis on assessing the diagnostic sensitivity, specificity and accuracy of CTA in detecting aneurysms compared with digital substraction angiography (DSA) and pointed out that the sensitivity, specificity and accuracy were high by using optimized acquisition and postprocessing protocols[14]-[17]. The purpose of our study was to assess the clinical applications of 16-row multislice CTA as the primary imaging technique in the surgical clipping and endovascular coiling of intracranial aneurysms.

## MATERIALS AND METHODS

### General data of subjects

Between May 2007 and May 2010, 195 (77 men, 118 women; age range of 24–84 years; mean age, 54 years) patients with 206 intracranial aneurysms underwent CTA examinations. One hundred and forty of these 195 patients had SAH, 21 patients had SAH and cerebroventricular haemorrhage (CVH), seven patients had SAH and intracerebral hematomas (IH), seven patients had SAH, IH and CVH, nine patients had IH, three patients had IH and CVH, one patient had CVH, four patients had headache, two patients had tumor and one patient had hydrencephaly.

### CTA acquisition

All CTA examinations were performed within 24 h after admission, and in most cases following unenhanced CT scan. The CTA study was performed with a 16-row multislice CT machine (Somaton Sensation 16, Siemens, Germany) with the following acquisition and reconstruction parameters: collimation 0.75 mm, gantry rotation time 0.5 sec, 120 kV, 250 mAs, postprocessing reconstruction 0.75 mm/0.4 mm. Contrast enhancement was provided by the intravenous antecubital administration of an 85-100 ml bolus of nonionic iodinated contrast material (XENETIX 350 mg I/ml, GUERBET, Roissy, France) at a 3 mL/sec flow rate. The contrast material was administered with a power injector (Medrad Stellant, Pa). An automatic fluoroscopic bolus-triggered technique determined the optimal timing of the data acquisition, the region of interest was placed at the internal carotid artery and scanning was started at 80 HU. CTA was initiated 15-25.8 sec (mean time 18.7 sec) after the start of an intravenous infusion. A caudocranial scanning direction was selected, covering the volume extending from the first cervical vertebra to the superior aspect of the frontal sinuses. In 11 patients with confusion or agitation or both, intravenous sedation was administered before CT angiographic examinations. When the CTA findings were considered sufficient for surgical planning, patients with mass IH underwent emergent surgical clipping without further diagnostic examinations.

### Image analysis

All CTA images were reviewed by two neuroradiologists (W. Chen and Y. Sun) independently. The reviewers had access to brief anonymous clinical details (on the radiology request cards) as well as to the anonymous plain CT examinations in order to reproduce the circumstances of routine clinical practice. The review of the CT angiograms was performed on a workstation (Siemens Wizard) to allow interactive reconstruction and interpretation, which has proved to be more accurate than an isolated review of hardcopy images[7]. The reviewer evaluated axial raw images, volume-rendering (VR) and maximum-intensity projection (MIP) images. CTA image reconstruction and interpretation lasted 10-15 min (mean time: 12 min) for each patient. The reviewer assessed the presence, shape (saccular or fusiform or irregular), orientation, neck, location of aneurysms, and the relationship of aneurysms to other structures such as bone and adjacent branch vessels. When the aneurysms were clipped, we emphasized evaluating the relationship of aneurysms to bone structures and the vascular images of different operative approaches by VR technique, and assessing the relationship of aneurysms to IH by MIP technique. When the aneurysms were coiled, we found the most clearly projectional angle of displaying the aneurysm neck by VR technique, and observed the relationship of aneurysms to the parent arteries and other branch vessels by MIP technique; the aneurysm dome (D) and neck (N) dimensions were measured at a selected projection on CTA images, which allowed the optimal demonstration of the neck of the aneurysm. The N/D ratio was calculated: narrow (N/D ratio < 0.50), intermediate (N/D ratio, 0.50-0.80) and broad (N/D ratio > 0.80). This procedure helped to determin whether stent implantation was needed; when stent-assisted endovascular coiling was performed, the width of the parent artery was measured on CTA images.

### Aneurysm treatment

Interventional neuroradiologist (W. Xing) and neurosurgeons (Y. Yang and Y. Peng) decided whether any target aneurysm was suitable for surgical clipping or endovascular coiling by using the 16-slice CT angiographic images, and endovascular coiling was the preferred treatment method. The following criteria were used to make the endovascular decision: an aneurysm was considered coilable if the aneurysm neck was considered narrow enough compared with the dome width to place coils within the aneurysm sack without coil protrusion into the parent artery, or the aneurysm neck was considered broad and stent implantation could prevent coil protrusion into the parent artery, and there were no vessel branches originating from the aneurysm above the neck level that might be occluded by the coil package. Endovascular treatment with Guglielmi detachable coils (GDC) was performed under general anesthesia. Combined stent implantation was used in broad-necked aneurysms, and the parent arteries were occluded with balloons or GDC in giant or distal aneurysms. If cerebral vasospasm was present in the endovascular procedure, papaverine was administered via the cerebral vasospasm artery. Surgical clipping was offered only if one of the following factors was present: first, the anatomy shown on the angiogram appeared unfavorable for endovascular therapy; second, the aneurysm was partially thrombosed; third, an IH was present with a mass effect; fourth, some patients could not afford the endovascular coiling, and surgical clipping was adopted[2]. Pterional craniotomies were performed for all clipped aneurysms. The titanium clips were used in the surgical treatment.

### Statistical analysis

Because it was well-recognized that DSA may occasionally fail to detect aneurysms, DSA alone was not considered as the gold standard in the study. We used conventional DSA or surgical findings or both as the ultimate reference standard against which the diagnostic accuracy of CTA and DSA was compared. Two-sided 95% (exact) confidence intervals based on binomial probabilities were calculated for each independent reader for each statistical parameter[18]. All statistical evaluation was carried out by Stata 9.2 for Windows (Stata Corp., USA). Comparisons between groups were made by Fisher exact test. Differences with a *P* value less than 0.05 were considered significant.

## RESULTS

**Fig. 1:**
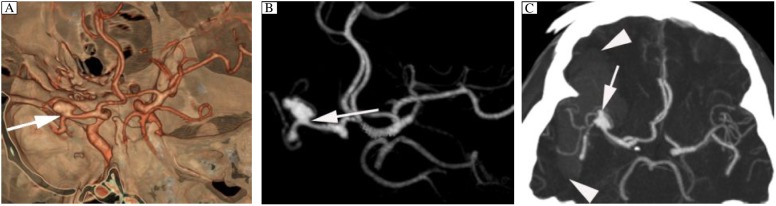
Right middle cerebral artery aneurysm in a 58-year-old man with SAH and intracranial hematoma (IH). A: VR image from CTA clearly display the relationship of the aneurysm to bone structures, adjacent branch vessels, and aneurysmal neck (arrow). B: MIP image from CTA clearly demonstrates the relationship of the aneurysm (arrow). C: Thin-MIP image from CTA clearly demonstrates the relationship of the aneurysm to IH (arrowhead), and the ruptured aneurysm has a small “nipple” (arrow). CTA: computed tomography angiography; MIP: maxium intensity projection; SAH: subarachnoid hemorrhage; VR: volume rendering.

**Fig. 2:**
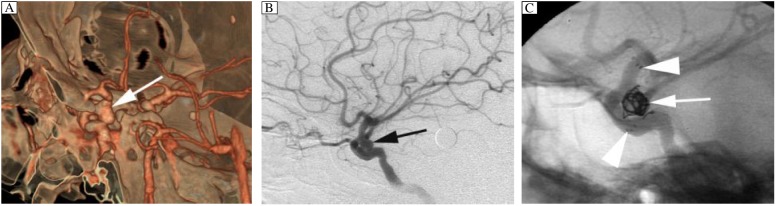
Right posterior communicating artery aneurysm in a 59-year-old woman with SAH. A: VR image from CTA clearly shows the broad-necked aneurysm (arrow), and indicates that the aneurysm should be coiled with a stent-assisted technique. B: the broad-necked aneurysm was proved by DSA images (arrow). C: The broad-necked aneurysm was treated with a stent implantation (arrowhead). SAH: subarachnoid hemorrhage; VR: volume rendering; CTA: computed tomography angiography; DSA: digital substraction angiography.

Three aneurysms were not depicted by CTA initially while visible on the CT angiographic images retrospectively. The primary reason for three missed aneurysms seemed to be the close proximity to bone and the small size of the aneurysm. Five aneurysms identified on CTA and confirmed at surgery were not clearly depicted at DSA. The false negative results of the five cases were directly due to acquisition of limited biplane projections and the small size of the aneurysms. Two aneurysms were not detected in the first CTA and DSA, but were clearly displayed in the repeated examination two weeks later. Retrospective review of CTA and DSA images showed that the two aneurysms probably had intrasaccular thrombus initially. The sensitivity of CTA and DSA in the detection of all aneurysms were 97.6% [95% confidence interval (CI): 94.4% to 99.2%] and 96.4% (95% CI: 92.8% to 98.6%), respectively, on a per-aneurysm basis. Although per-aneurysm statistical results of CTA differed from that of DSA, there was no statistically significant difference in sensitivity between 16-slice CTA and DSA (*P* = 0.73). One hundred and eighty-four patients (94%) harbored one aneurysm (***Fig. 1*** and ***Fig. 2***), and eleven patients (6%) had two aneurysms. The most common location for all the aneurysms was the posterior communicating artery (PCoA: 32%), and the second most common location was anterior communicating artery (31%). One hundred and sixty-one aneurysms (78%) were saccular in shape, 30 (15%) were irregular and 15 (7%) fusiform. The mean aneurysm size was 6.2 mm (range, 1.9-21.4 mm). The average aneurysm neck size was 3.5 mm (range, 1.2-10.0 mm), the average aneurysm dome size was 4.7 mm (range, 1.4-16.1 mm), and the mean N/D ratio was 0.83 (range, 0.20-1.75).

In the surgical group with 50 aneurysms, the five missed aneurysms at DSA and the nine aneurysms with mass IH (28%) were successfully treated based on 16-slice CTA as the only preoperative investigation (***Fig. 1***) while the other 36 aneurysms (72%) were clipped mainly on the basis of the 3D CTA images. Forty-eight aneurysms were treated with a single clip and two with two clips. Sixteen-slice CTA depicted the relationship of the aneurysms, bone structures and IH, and provided good visualization of the dome and the neck of the aneurysms as well as of the adjacent arterial branches (***Fig. 1***), which guided the surgical treatment of the aneurysms. The surgical findings were well correlated with surgical views generated with the CT angiographic data in all clipped aneurysms. All aneurysms were deemed completely occluded, and all parent arteries were thought to be patent during surgical clipping.

In the endovascular group with 156 aneurysms, most of the coiled aneurysms simultaneously underwent DSA and endovascular therapy. Sixteen-slice CT angiographic images could provide essential preoperative information, such as the shape, dimension and location of the aneurysms, the parent artery and the N/D ratio, which could assist in guiding the catheter direction and finding the clearly projectional angle, and decide whether stent implantation was needed (***Fig. 2***). In the study, 16-slice CTA images displayed 15 broad-necked aneurysms, and indicated that it was difficult for them to undergo coiling without a stent-assisted technique. Fourteen of the 15 aneurysms were coiled successfully with stent assistance in the endovascular therapy (***Fig. 2***). However, one aneurysm failed to be coiled initially because a stent implantation was not considered by the interventional neuroradiologist, and the aneurysm underwent a successful therapy with an endovascular stent nine days later.

In addition, usually one of the multiple aneurysms is the ruptured aneurysm, and CTA images can determine the ruptured aneurysms in some patients because most ruptured aneurysms have one or two small “nipples”. In the 11 multiple aneurysms of the study, eight ruptured aneurysms were decided by the shape of the aneurysms, and were firstly treated. These patients with multiple aneurysms had a good recovery after the treatments.

## DISCUSSION

SAH due to ruptured aneurysms often results in poor prognosis. Mortality is high among patients with this condition. Most of the patients die of rebleeding in additional to the initial IH, and appropriate treatment should be performed to minimize rebleeding[2],[3]. Therefore, it is very important to promptly diagnose and treat the ruptured aneurysms. In our hospital, 16-slice CTA has been employed as a first-line imaging modality in screening patients with SAH possibly caused by ruptured aneurysm. Compared with DSA, 16-slice CTA is simpler and quicker to organize and perform. In the emergency setting, 16-slice CTA can be performed immediately after a diagnosis of SAH has been given based on a routine plain CT scan of the brain. Confused, irritable or uncooperative patients need no more than a short-acting sedative to complete CTA, and a complete diagnostic workup can be performed without general anesthesia in most cases, which is often required during an emergency angiographic workup[19]-[21]. Moreover, we found that the sensitivity of CTA in the detection of all aneurysms was high, and there was no statistically significant difference in sensitivity between 16-slice CTA and DSA (*P* > 0.05). In our study, 16-slice CTA detected nine ruptured aneurysms with mass IH, and the preoperative workup and therapeutic decisions were based on 16-slice CT angiogram findings alone for these aneurysms. When these patients were in the process of preoperative preparation, CT reconstructions were performed simultaneously. Sixteen-slice CT images were displayed in the operating room and provided much valuable information, which guided the surgical treatment of these aneurysms. These aneurysms were successfully treated by means of surgical clipping, and the IH was removed at the same time (***Fig. 1***). Therefore, the pretreatment evaluation time was shortened critically, and it would increase the survival rate of the patients.

There have been important technical advances including smaller catheters, hydrophylic guide wires and digital imaging systems[22]. However, the technical advances in cerebral angiography have not overcome the patient-related risk factors associated with neurologic complications. The risk of neurologic complications increases with age. Cardiovascular disease and fluoroscopic times longer than 10 min are independent predictors of risk. These findings substantiate the argument that patients at a higher risk should undergo minimally invasive imaging of the craniocervical vessels and that catheter angiography should be avoided. In patients without these risk factors, neurologic complications still occur and, therefore, the indications for catheter angiography should be limited. In our study, 16-slice CTA detected nine ruptured aneurysms with large IH, and the therapeutic decisions were based on 16-slice CTA findings alone for these aneurysms and purely diagnostic DSA was avoided.

In the surgical clipping of aneurysms, sometimes there are blind corners in an aneurysm due to the difficulty in gaining proximal artery control and a sufficient surgical field, which may lead to unsatisfactory results. Therefore, a detailed understanding of aneurysm morphology and the adjacent vasculature is necessary to perform a safe and successful operation[23]-[26]. Many reports pointed out that it seemed safe and effective to make decisions regarding surgical treatment on the basis of CTA alone in the majority of patients with ruptured or unruptured aneurysms[13],[23],[24],[27]. Dehdashti *et al*.[13] considered that spiral CTA allowed reliable pretreatment planning for the majority of cases of aneurysmal SAH and diminished the pretreatment evaluation time critically. Pechlivanis *et al*.[24] reported a careful analysis showly that most aneurysms could be operated solely on four-slice CTA data. Their results showed the value of the anatomic information provided by CTA alone. In our study, 50 of 206 aneurysms underwent surgery, 16-slice CTA images allow us to observe aneurysms, the relationship of aneurysms to bone structures and adjacent branch vessels, and the surgical findings correlated well with the CTA findings. Especially in the five missed aneurysms at DSA and the nine aneurysms with mass IH, their surgical treatment planning were based on CTA alone, and these aneurysms were safely treated. Therefore, the 16-slice CTA images were useful references for the surgical treatment of aneurysms (***Fig. 1***). In addition, CTA is also a valuable noninvasive diagnostic modality for the assessment in patients after aneurysm clipping[28]-[30].

An accurate imaging study is also very important to assist DSA and endovascular treatment of aneurysms[31]-[34]. Papke *et al*.[19] had observed whether 16-slice CTA could provide sufficient diagnostic information to guide endovascular treatment, and they reported that the possibility of coil embolization was correctly assessed with 16-slice CTA in 69 (93%) of 74 target aneurysms for acute occlusive treatment. If 16-slice CTA depicts the ruptured aneurysm, a diagnostic-only DSA without the possibility to initiate endovascular treatment in the same session could be avoided, especially in patients with aneurysms that appeared to be good candidates for coil placement at CTA. In our study, the interventional neuroradiologists could explain to the patients' families the purpose and significance of the aneurysm treatment based on CTA results because most aneurysms have been definitely diagnosed by CTA images before the endovascular therapy. Therefore, most of the coiled patients underwent DSA and endovascular therapy at the same time. However, three aneurysms were not initially detected by CTA, and could not simultaneously undergo DSA and endovascular treatment because the interventional neuroradiologists could not talk with the patients' families about endovascular therapy for the aneurysms. Consequently, the three patients suffered more both physically and financially. In addition, it was sometimes difficult to evaluate the relationship between the aneurysm and parent artery with DSA (***Fig. 2***). Sixteen-slice CT angiographic images could provide detailed information, such as the shape, dimension and location of aneurysms, the relationship of the aneurysm to the parent artery, the N/D ratio and the most clearly projectional angle, which could guide DSA and endovascular treatment of aneurysms, especially for broad-neck and distal aneurysms (***Fig. 2***). The angiographic numbers and the contrast material volumes were obviously decreased in the DSA procedure. Broad-neck aneurysms were coiled with stent-assistance and all aneurysms underwent successful occlusive treatment. Therefore, 16-slice CTA has assisted DSA and endovascular coiling of the aneurysms.

In conclusion, sixteen-slice CTA image information has been shown to determine the choice of aneurysm therapy and assist the surgical and endovascular treatment of aneurysms. The study has confirmed the clinical value of 16-slice CTA as the primary imaging technique in the surgical clipping and endovascular coiling of intracranial aneurysms.
